# Training Improves the Capacity of Visual Working Memory When It Is Adaptive, Individualized, and Targeted

**DOI:** 10.1371/journal.pone.0121702

**Published:** 2015-04-02

**Authors:** Eunsam Shin, Hunjae Lee, Sang-Ah Yoo, Sang Chul Chong

**Affiliations:** 1 Center for Cognitive Science, Yonsei University, Seoul, South Korea; 2 Graduate Program in Cognitive Science, Yonsei University, Seoul, South Korea; 3 Department of Psychology, Yonsei University, Seoul, South Korea; Centre de Neuroscience Cognitive, FRANCE

## Abstract

The current study investigated whether training improves the capacity of visual working memory using individualized adaptive training methods. Two groups of participants were trained for two targeted processes, filtering and consolidation. Before and after the training, the participants, including those with no training, performed a lateralized change detection task in which one side of the visual display had to be selected and the other side ignored. Across ten-day training sessions, the participants performed two modified versions of the lateralized change detection task. The number of distractors and duration of the consolidation period were adjusted individually to increase the task difficulty of the filtering and consolidation training, respectively. Results showed that the degree of improvement shown during the training was positively correlated with the increase in memory capacity, and training-induced benefits were most evident for larger set sizes in the filtering training group. These results suggest that visual working memory training is effective, especially when it is adaptive, individualized, and targeted.

## Introduction

We live in a cluttered world which is richly populated with objects and ongoing events. Visual working memory (VWM) enables us to maintain the continuity of our visual experience by linking past information with the current flow of target information, and is essential for performing various visual tasks in this cluttered environment. Despite such importance, the visual system can retain only a small number of items in VWM [1, 2]. This has led researchers to find ways to increase its capacity, typically through training.

Table 1 summarizes past VWM studies in which the effects of training on VWM performance were tested. The summary is presented in chronological order, from the most recent years on. Earlier studies [3–5] investigated whether learning memory arrays from repeated presentations could lead to a better performance (e.g., an increase in accuracy). More recent studies have begun to examine the impact of training [6–8] or visual expertise [8–10] on the memory capacity. It has been reported that gaining visual expertise on an object category (e.g., cars, letters) increased the memory capacity for that category. However, training with VWM tasks has not yielded consistent memory capacity benefits.

**Table 1:** VWM studies in which training were investigated in humans.

Study	Task		Period	Stimulus	Result
Arend & Zimmer (2012)	Train	*Adaptive* multiple object tracking (distractor addition)	10 sessions for 14 days (5 hrs.)	Colored squares and rectangles	
	Test	Change detection in a divided-field with distractors		Black circles	No transfer effects
Zimmer et al. (2012)	Train	*Adaptive* change-detection (duration of memory arrays and set size)	12 sessions over 4 weeks	Chinese characters and artificial patterns	
	Test	Change-detection with one test stimulus		Trained and untrained Chinese characters and artificial patterns	Item-specific positive effects
Berry et al. (2010)	Train	*Adaptive* motion discrimination task (speed of motion and interstimulus interval)	3–5 sessions / week for 3–5 weeks (10 hrs.)	Extracted or contracted Gabor patterns	
	Test	Change detection in motion directions		Moving dot kinetograms	Positive transfer effects
Moore et al. (2006)	Train	*Adaptive* match-to-sample task (duration and stimulus discriminability)	7 sessions over 10 days (10.5 hrs.)	Novel polygons	
	Test	Match-to-sample		Trained and untrained polygons	Increased brain activity (memory capacity not assessed)
Chen et al. (2006)	Train	Change detection with two test stimuli	320 trials	Random polygons	
	Test	Change detection with two test stimuli		Random polygons	No familiarity effects
Olson et al. (2005)	Train	Change detection with one item missing in a test array. Some memory arrays presented repeatedly.	32 repetitions per memory array	Green squares	
	Test	Change detection in random or predicted locations		Green squares	Positive effects only in predicted target locations
Olson & Jiang (2004)	Train	Change detection in cued locations.Some memory arrays presented repeatedly	24 repetitions per memory array	Green squares and novel shapes	
	Test	Change detection while being trained		Green squares and novel shapes	No effects of repeated memory arrays
Olsen et al. (2004)	Train	*Adaptive* visuospatial working memory tasks (set size)	90 trials / day (35–45 min.) for 5 weeks	Colored circles	
	Test	The serial positioning task, neuropsychological tests		Colored circles	Increased brain activity and positive transfer effects

These inconsistent results may have been caused by the differences in training methods and contents. As shown in the table, the studies using adaptive methods over extended periods of time [7, 8, 11, 12] have tended to show positive training effects, with one exception [6]. Note that the parameters used in the adaptive training are indicated in parentheses in the table. For instance, Zimmer et al. [8] adjusted the duration of the memory arrays and the set size of the memory items to increase task difficulty as the training progressed. Thus, it appears that adaptive training effectively enhances VWM capacity, as discussed in the context of general WM [13]. Furthermore, the efficacy of the adaptive training seems to be enhanced when the adjustment of task difficulty is individually tailored (e.g., [7, 11, 14]). Given that individuals have different learning curves in terms of start points and slopes (i.e., learning speeds), it is reasonable to hypothesize that an adaptive training combined with an individually tailored approach would increase the likelihood of enhancing VWM capacity.

Unlike earlier studies, which focused on familiarization with memory items or their spatial arrays [3–5], more recent studies have begun to focus on targeting specific component processes, such as distractor filtering [6] or stimulus encoding [11]. Using multiple object tracking, Arend and Zimmer [6] trained participants for 10 sessions over 14 days to enhance their filtering abilities, with the idea that enhanced filtering efficiency would benefit their VWM capacity. Before and after the multiple object tracking training, participants performed the lateralized change detection task [15] in which one side of the visual field had to be attended to while the other had to be ignored, and distractor items were presented with target items in some conditions. Although the training task increased the filtering efficiency of the participants, this increase was not transferred to enhancing VWM capacity in the change detection task.

Arend and Zimmer [6] is an exemplar of VWM training study for us, as (a) it directly tested the effects of training on the VWM capacity; (b) it used an adaptively designed training method targeting filtering efficiency; and yet (c) it failed to show that an increased filtering efficiency transferred to enhancing the capacity of VWM. This lack of evidence for the transfer of the benefit may have been generated from the different filtering mechanisms operated in the two tasks (p. 2387, [6]). Selective attention would have been a more relevant process than sustained attention to target in the lateralized change detection task. In other words, a form of training aimed at developing selective attention skills would have produced a transfer of benefit in the lateralized change detection task.

**Fig 1:**
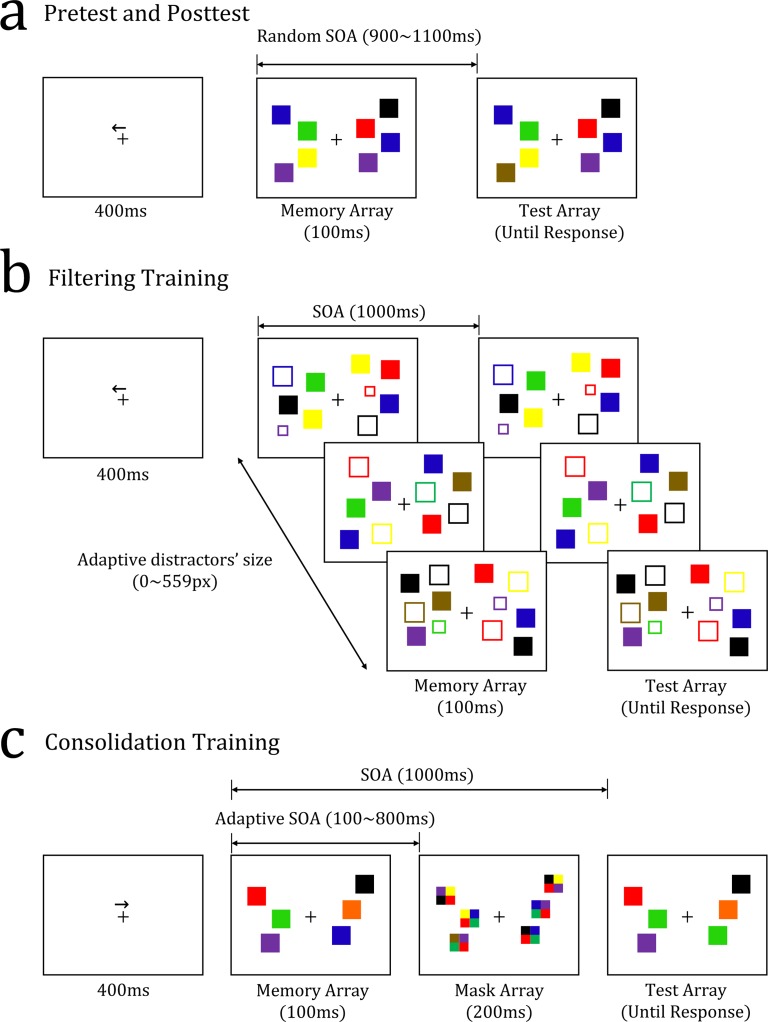
Examples of trial sequences in the pretest and posttest (a), the filtering training (b), and the consolidation training (c). The arrows indicate which memory array out of the two sides had to be remembered. The color-filled squares represented the target items to be remembered, and the color-outlined squares the distractor items to be ignored (b). The task was to judge whether the memory array and the test array were the same or different. (b) and (c) show examples of correct answers, same and different, respectively. Finally, the SOA represents the stimulus onset asynchrony, which includes the duration of the memory array.

The current study adopted individually-tailored adaptive training methods with two target processes, filtering and consolidation. A lateralized change detection task with no distractors [16] was used as a test task (Fig 1a). Two modified lateralized change detection tasks [15, 17] were used as training tasks. One training task challenged the participants’ filtering ability by adjusting the number of distractor items presented with target items (Fig 1b). The other task challenged the participants’ consolidation ability by varying the consolidation durations (Fig 1c). The test task has been widely used to estimate VWM capacity [18. 19]. The key feature of this task is that it requires the participant to attend selectively to one side over the other. Thus, a greater transfer of benefit (if any) should arise from the filtering training than from the consolidation training, as the filtering training is likely to improve selective attention, part of the general WM component process, and, in turn, to develop effective encoding and maintenance skills. Instead, the consolidation training is expected to improve quick skills of item registration and consolidation, which are distinct from filtering skills. In this sense, the participants who received the filtering training served as an experimental group, and those who received the consolidation training served as an active control group. As the active control group underwent a procedure and a training time closely matched with those of the experimental group, the existence of this active control group prevented the training results from being inflated in positive directions [20].

If our training were to produce positive effects on VWM capacity, the following results were expected. First, VWM capacity would increase in the trained participants compared to the untrained participants (inactive control group). Second, the training effects would differ depending on the processes targeted during the training. Given that the test task required attention shift and item selection processes, the idea of process-specific improvement predicted that the participants who received the filtering training would show more benefits than those who underwent the consolidation training. To preview our results, the overall training effects were positive: (a) VWM capacity significantly increased in the filtering group, particularly for larger set sizes; (b) the extent to which the participants’ VWM capacities increased was positively correlated with the improvements displayed by the participants during the training.

## Materials and Methods

### Participants

Sixty-two adults (42 females; mean age = 24.48, *SD* = 4.21) were recruited from the Yonsei University community. We chose this number of participants based on Arend and Zimmer’s study [6], in which 20 participants were selected for each group. The participants had normal or corrected-to-normal visual acuity, and normal color vision. The experimental protocol was approved by the Institutional Review Board of Yonsei University, and all participants gave their signed informed consent. As one participant gave up after one training session, the original sample size 62 was reduced to 61 (20 in the filtering group, 21 in the consolidation group, and 20 in the control group) at the completion of the study.

### Design

The participants were randomly assigned to one of the three groups. Those in the filtering and consolidation groups completed a pretest, 10 training sessions, and a posttest. All the sessions, including the pre- and posttests, were held on separate days. The control group only partook in the pre- and posttests, with the same 10-session interval between them. The participants were not informed of which group they had been assigned to. An average of 15.41 calendar days was taken for each participant to complete the study. After completion, the participants received 10,000 KRW for their participation in each pre-/posttest, and 8,000 KRW per training session. The participants in both training groups received an additional 2,000 KRW incentive when their performance improved from one training session to the next. 1,000 KRW is approximately equivalent to 1 USD.

### Pretest and Posttest

The pretest and posttest were run to evaluate the effects of the training on VWM capacity. During the tests, the participants performed a lateralized change detection task which required the encoding of stimuli in a divided visual field [16]. In this task, each trial began with a left- or right-pointing arrow which was shown for 400 ms just above a central fixation cross which remained throughout the experiment. This arrow indicated the visual field to which the participants had to attend. A memory array consisting of colored squares was then presented for 100 ms (see Fig 1a). Following a randomly selected retention interval between 800 and 1000 ms, a test array showing items either identical to the memory array (same) or different by one colored item (different) was presented until a response was made. The ratio of the same to different trials was 50:50.

The participants were asked to determine whether the memory and test arrays in the attended field were the same or different, and to respond with one hand by pressing one of two keys on a computer keyboard. Sound feedback was given whenever their responses were incorrect. The participants were told that fixating on the central cross was the best strategy for performing the task and were encouraged to maintain their fixation throughout the experiment. The participants completed 40 practice trials prior to the actual test.

All stimuli were presented on a gray background (14.43 cd/m^2^) and were displayed within two 3.5° × 7° subtending imaginary rectangular regions, whose centers were placed 3° to the left and right sides of the fixation cross. Two, four, five, six, or seven colored squares (0.7° × 0.7°) were simultaneously presented in each visual field. The colors of the squares were randomly selected from a set of 9 highly discernible colors (red, green, blue, yellow, violet, brown, orange, black, and white), with the constraint that same-colored squares could not appear within each visual field. The stimuli were randomly placed, with the constraint that the center-to-center distance between the squares should be at least 1°. There were 20 blocks in total and each block consisted of 20 trials (5 set sizes × 2 visual fields × change or no change). The order of the trials was randomized within each block. A sixty-second break was provided every 40 trials and it took approximately 40 minutes for each participant to complete the test.

### Training

The filtering and consolidation training groups performed variants of the color change detection task used in the pre- and posttests. The tasks were varied in the following ways: for the filtering training, we added outlined squares of different colors (i.e., distractors) to the memory and test arrays (Fig 1b). Because VWM was only tested for the color-filled squares (i.e., targets), the distractors were presented to generate perceptual interference. For the consolidation training, we added a mask display after presentation of the memory array. This mask display was presented to make it harder to encode and consolidate the memory items (Fig 1c).

In each training session, we employed an adaptive staircase procedure [21] in which two staircases were alternately chosen in each trial to prevent the participants from anticipating the difficulty of the next trial. Each staircase was terminated after 20 reversals. During the filtering training, the number of distractors increased or decreased depending on the response accuracy. In doing so, the sum of all the distractors’ sides in an array was generated in every trial, and was increased after two consecutive correct responses and decreased after a single incorrect response (i.e., 2 up and 1 down). This was used as our strategy to adjust the number of distractors and, consequently, to manipulate the difficulty of filtering the distractor items.

Given that the side of the square stimulus was 43 pixels long, the number of distractors present in an array multiplied by the square of 43 would have represented the sum of the distractors’ areas in each trial. However, as the four sides of a square have the same length, we only report the amount of pixels that composed one side in order to describe how the number of distractors was changed. Any length from 43 (43 × 1) to 129 (43 × 3) pixels was chosen for the initial value of each staircase in the first session, and 16 pixels were then added to or subtracted from each square side, according to the 2-up-and-1-down staircase. This method allowed the remainder of the sum of the squares’ sides divided by 43 pixels to be unequal to 0, and, therefore, for a smaller distractor to be generated with the remaining pixels (as a cell divides). For example, 59 pixels generated two distractors: one square with a 43-pixel side and one smaller square with a 16-pixel side (see Fig 1b). The step size was changed to 8 pixels after 8 reversals. The distractors presented in the memory array were shown again in the test array.

In the consolidation training, the time interval between the memory array and the mask display decreased or increased depending on the response accuracy. More specifically, masks were 2×2 checkerboard squares (0.8° × 0.8°) filled with four randomly-selected colors among the 9 colors used in the pre- and posttests. These masks appeared in the same locations as the memory items. The memory-mask interval decreased after two consecutive correct responses and increased after a single incorrect response (2-down and 1-up). This interval was changed by a time of 59 ms (5 frames). For each staircase, the very first interval was randomly determined between 275 and 625 ms in the first session. The step size was changed to 24 ms (2 frames) after 8 reversals. The memory array was always presented for 100 ms as in the consolidation group, and the interval between the memory and test arrays was always 900 ms. Thus, the interval between the mask offset and the test-array onset depended on the memory-mask interval in that trial.

The completion of one session provided the initial values of the sum of all the distractors’ sides and the memory-mask interval for the next session of the filtering and consolidation trainings, respectively. The values (either the sums or the intervals) that had been generated in the last 4 reversals were averaged for each staircase, resulting in two separate staircase means. Note that these means provided the thresholds at which the participants maintained similar levels of task performance across different sessions [21]. In the next session, two values within the range of the two separate means were randomly selected and used as the initial values of the two staircases. The completion of the two staircases, which took approximately 20 minutes, terminated each session. At the beginning and end of each session, the participants were informed of their performance (i.e., the average of the two staircase means) and their accumulated monetary rewards. The stimuli and procedure used in the training were the same as in the pre- and posttest sessions, except for the following: (a) the set sizes were 3, 5, 6, and 7 instead of 2, 4, 5, 6, and 7; (b) no practice trials were given; and (c) a resting time was given every 45 trials.

## Results

Training effects were assessed by estimating VWM capacity (denoted *H*) in each set size for each participant in the pre- and posttest sessions, following a formula developed by Pashler ([18], also see [19]): memory capacity = set size x (hit rate—false alarm rate)/(1—false alarm rate). Four participants showed very low memory capacities (ranging from -0.29 to 0.88) in the set sizes 6 and 7 conditions. These estimates were distinct from those obtained from the same participants in the other set size conditions as well as those of the other participants, and created anomalies in the data set. In addition, one participant demonstrated a very poor pretest performance (43% accuracy even in the set size 2). To ensure the integrity of the data set, these five participants were excluded from further analyses, leaving 56 participants (19, 19, and 18 in the filtering, consolidation, and control groups, respectively) to be entered into the statistical analysis.

**Fig 2:**
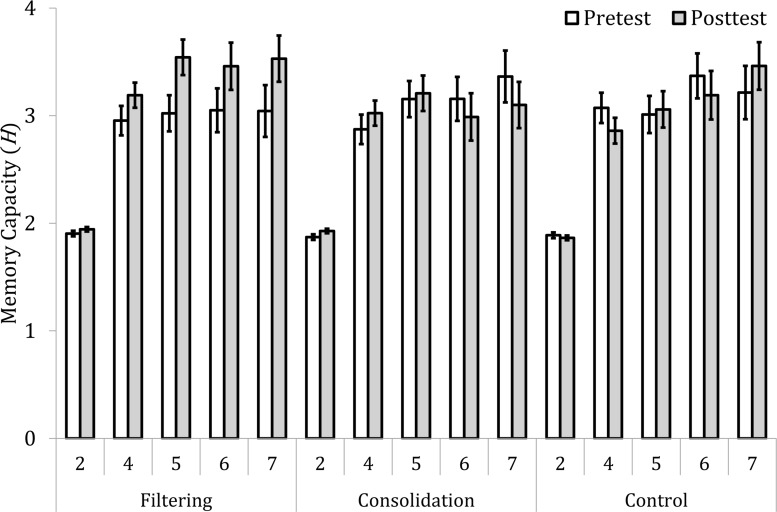
Memory capacity *H* in the five set sizes across the three groups in the pretest and posttest.

Fig 2 shows the memory capacity across the five set sizes in the three groups at the pretest and the posttest. We first tested whether there were group differences in the VWM capacity before the training. The average *H*s of the filtering, consolidation, and control groups were 2.80, 2.88, and 2.91, respectively, and were not significantly different from one another, *F*(2, 53) = .217, *p* = .806, as revealed by a 3 (group: filtering, consolidation, and control) × 5 (set size: 2, 4, 5, 6, and 7) mixed factorial analysis of variance (ANOVA) with repeated measures on the second factor. The memory capacity significantly increased as the set size became larger, *F*(4, 212) = 56.851, *p* <. 001, *η*_*p*_^*2*^ = .518, but this increase did not interact with the group, *F*(8, 212) = .781, *p* = .620.

Second, we tested whether there were positive training effects. The *H* measure of the items held in VWM was submitted to a 3 (group: filtering, consolidation, and control) × 2 (test: pretest, posttest) × 5 (set size: 2, 4, 5, 6, and 7) mixed factorial ANOVA with repeated measures on the second and third factors. The memory capacity significantly differed by set size, *F*(4, 212) = 97.820, *p* <. 001, *η*_*p*_^*2*^ = .649, but did not differ by group, *F*(2, 53) = .181, *p* = .835, or by test, *F*(1, 53) = 2.839, *p* = .098. None of the two-way interactions were significant (*ps* >. 092), except for a group × test interaction, *F*(2, 53) = 4.960, *p* = .011, *η*_*p*_^*2*^ = .158.

To investigate the nature of this significant interaction, we compared the capacity *H* obtained in the pretest with that in the posttest for each group. The filtering group showed a significant improvement (from 2.80 in the pretest to 3.13 in the posttest), *F*(1, 18) = 11.699, *p* = .003, *η*_*p*_^*2*^ = .394, indicative of the efficacy of the filtering training. The consolidation group, however, did not show a significant capacity improvement (from 2.88 to 2.85), *F*(1, 18) = .113, *p* = .741. As expected, the control group did not show any significant difference between the pretest (2.91) and posttest (2.89), *F*(1, 17) = .084, *p* = .775. We defined a training gain as the *H* difference between the pretest and the posttest (with all set sizes being collapsed). Using independent-samples *t* tests, the training gain was compared between two groups. The filtering group showed a significantly higher gain than the consolidation group, *t*(36) = 2.613, *p* = .013, and also than the control group, *t*(35) = 2.790, *p* = .008. The consolidation group was not significantly different from the control group *t*(35) = .079, *p* = .938.

A three-way (group × test × set size) interaction was marginally significant, *F*(8, 212) = 1.814, *p* = .076. This trend was further analyzed using the training gain for each set size. The training gain was compared between the two different groups as the group × test interaction was examined above. The comparison between the filtering and control groups showed that the training gain was significantly higher in the filtering group than in the control group for the set sizes 4, 5, and 6, *ts*(35) > 2.060, *ps* < 0.048. Moreover, a significantly higher gain was found in the consolidation group, compared to the control group, for the set size 2 only, *t*(35) = 2.038, *p* = .049. Finally, the filtering group showed a significantly higher training gain than the consolidation group for the set sizes 5 and 7, both *ts*(36) > 2.274, *ps* <. 030. The set size 6 was marginally significant, *t*(36) = 1.757, *p* = .087. These results suggest that although both training methods improved the capacity of the VWM, the filtering training was more effective than the consolidation training.

**Fig 3:**
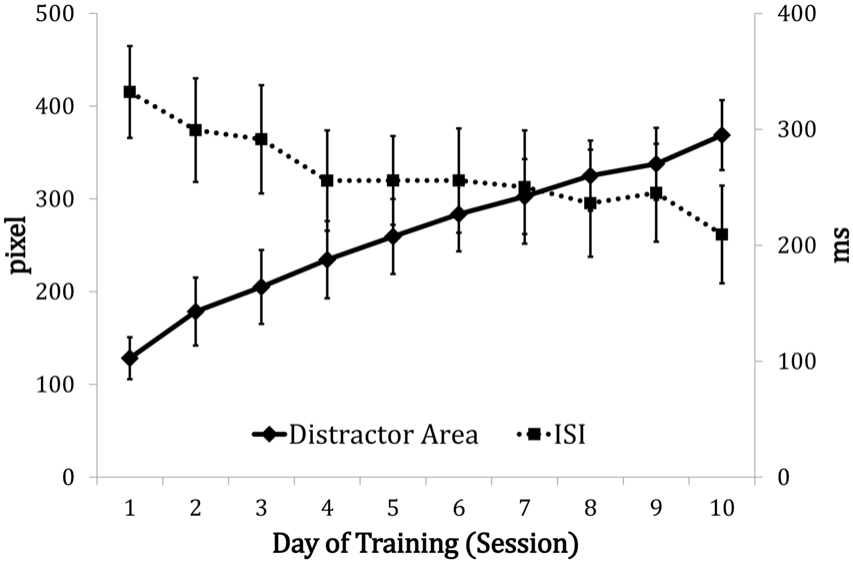
Thresholds (expressed in pixels and in milliseconds, ms) shown in both the filtering and consolidation groups across the ten training sessions. The distractor areas show the threshold changes in the filtering training, and the ISIs show the threshold changes in the consolidation training. The error bars indicate the standard error of the mean.

We then examined the changes in the filtering and consolidation groups over the 10 training sessions (Fig 3). In the filtering group, the maximal pixel size of the distractors (i.e., the threshold of distractor filtering) significantly increased, from 128 pixels (about 2.98 distractors) in the first session to 369 pixels (about 8.58 distractors) in the last session *F*(9, 162) = 27.297, *p* <. 001, *η*_*p*_^*2*^ = .603. This increase suggests that the participants successfully filtered out more distractors after receiving the training, while their change detection performance remained similar. In the consolidation group, the minimal memory-mask interval (i.e., the threshold of consolidation interval) significantly decreased, from 332 ms in the first session to 209 ms in the last session, *F*(9, 162) = 2.085, *p* = .034, *η*_*p*_^*2*^ = .104, suggesting that shorter consolidation intervals were needed to maintain similar levels of task performance after the training.

**Fig 4:**
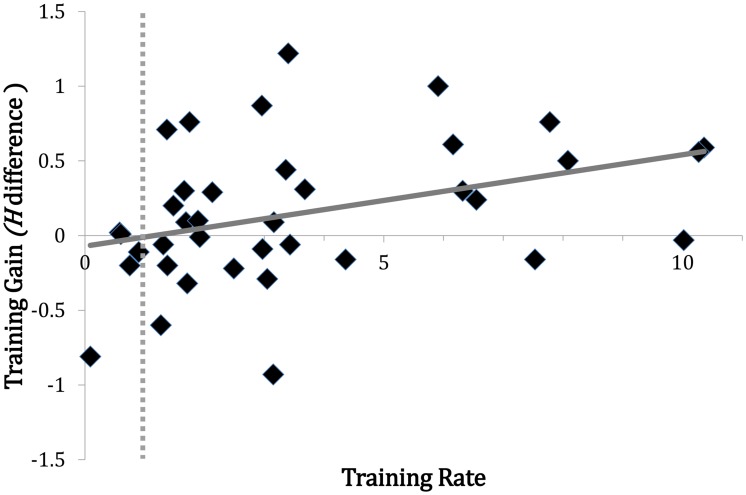
Correlation between training gains and threshold improvements across the training sessions. Tie point 1 (represented by the vertical dotted line) indicates the point at which the threshold of the first session was the same as that of the last session, showing no learning from the training. The error bars indicate the standard error of the mean.

We reported that the degree of increase in VWM capacity was different between the two training groups. The two groups also showed significant changes which indicated that some degree of learning had occurred with the increasing number of sessions. This learning effect was assessed in the following ways. First, we calculated the ratio of the first to last threshold. For the filtering group, the last threshold was divided by the first threshold. For the consolidation group, the first threshold was divided by the last threshold. This ratio was then correlated with the training gain. Fig 4 shows that the ratio was significantly correlated with the training gain in all trained participants, *r* = .373, *p* = .021. When these participants were separated by group, neither the filtering nor the consolidation group yielded significant correlations (*r* = .304, *p* = .206 and *r* = .406, *p* = .085, respectively). These results led us to further examine the set size effects in each training group. A significant correlation was found for set size 4 in the filtering group, *r* = .517, *p* = .023, and for set sizes 2 and 6 in the consolidation group (*r* = .545, *p* = .016 and *r* = .481, *p* = .037, respectively).

## Discussion

In the past years, a handful of VWM training studies have been conducted in various ways and with different focuses. However, very few of those studies have directly tested whether training enhances VWM capacity [6, 8]. The current study therefore tested the impact of training on VWM capacity, following the recent trends (adaptively designed training) in the WM training regimes. Across ten-day sessions, two groups of participants performed different tasks designed to improve specific cognitive skills—target selection and distractor inhibition in the filtering training group, and item registration and consolidation in the consolidation training group. Participants were continuously challenged as their performance improved, with the level of task difficulty tailored to each participant. Presumably, good control of attention is important to perform the lateralized change detection task from which the memory capacity was estimated. Thus, the filtering training served as the experimental group, and the consolidation acted as the active control group.

Our adaptive, individualized, and targeted training interventions yielded positive overall results: (a) the participants in the filtering group displayed significantly increased VWM capacities compared to those in the consolidation and control groups; (b) this capacity increase in the filtering group was significantly higher for the larger set sizes (4, 5, 6, and 7), in comparison to the consolidation and control groups; (c) compared to the control group, the consolidation group showed a capacity increase in the small set size 2 condition; (d) both the filtering and consolidation groups showed significant improvements (assessed by the threshold changes) over the course of the training sessions; (e) this improvement was correlated with the increase in capacity; and, more specifically, (f) this correlated relationship was found to be significant for the set size 4 in the filtering group and for the set sizes 2 and 6 in the consolidation group. These results indicate that the participants in both the filtering and consolidation groups had learned the targeted skills during the training period. However, the filtering training was more effective than the consolidation training in enhancing VWM capacity. Nonetheless, the results suggest that a transfer of the benefit occurred in the two training groups, albeit to different degrees. The benefit from the filtering training was transferred to larger set sizes, and the benefit from the consolidation training was transferred to a small set size.

Although the consolidation training showed a significant correlation between threshold change and training gain in the set size 6, it is hard to conclude that the benefit from this training was transferred to a larger set size. This significant correlation merely reflects the tendency that participants who showed greater performance improvement during the training also showed higher capacity increase in this particular set size condition after the training. It does not necessarily indicate that the skills learned during the training were transferred to those required for performing the lateralized change detection task. It is clear that this result helps understand training effects better. However, the result itself cannot stand alone as evidence of transfer of benefit without the corroboration from other test results (e.g., a significant gain difference in the set size 6 between the consolidation and control groups).

As expected, the filtering training generated the most positive effects on VWM capacity. The lateralized change detection task requires participants to selectively process one side of a visual display while ignoring the other. A good command of item selection and inhibition would be the gateway to efficient encoding, and continuing attention control should lead to good maintenance of the encoded items. Thus, as a key to maximizing the VWM storage [15, 22–24], good filtering skills were developed while performing the modified training task, and the benefit may have been transferred to selective attention in the divided field task. This transfer effect was more evident in the larger set sizes than the smaller ones, suggesting that the filtering training was particularly effective for larger memory loads (i.e., more difficult conditions).

The consolidation training also demonstrated some limited effects on VWM capacity. In particular, the set size 2 condition was associated with an increased memory capacity and with a significant correlation between the performance improvement and the capacity increase. The origin of this limited transfer effect is not obvious. If the consolidation training was indeed effective in increasing the memory capacity, some possibilities can be considered. Using a full-field change detection task, Vogel and colleagues [17] estimated that the rate of consolidation was about 50 ms per item. According to this estimation, set sizes 2 and 4 would require approximately 100 and 200 ms each. In the current study, the minimal memory-mask interval (i.e., the threshold of consolidation) was 209 ms in the final session. This interval is sufficiently longer than the 100 ms and is very close to the 200 ms estimation. This may mean that the consolidation training decreased the time needed for item registration and consolidation, to the extent that the set size 2 condition received benefits from the training, but the set sizes larger than 2 (i.e., 4, 5, 6, and 7) came short of receiving them. Other studies have also estimated the rate of consolidation [25–27] and have provided a wide range of estimates. Nonetheless, we discussed Vogel et al. [17] in particular because our consolidation training task was designed based on this study. In any case, the exact upper bound of the rate of consolidation is unknown. If there is a minimal time required to consolidate each item, any good training effect cannot go beyond this limit. Furthermore, even if the participants were able to register and consolidate larger set sizes within a short time, the fidelity of the memory representations may not have been high, assuming that the resources allocated to each item were limited. Hence, low-fidelity representations may have contributed to the lack of transfer effects for the larger set sizes.

It is important to note that our training interventions targeted specific processes and did not require the participants to repeat the test task during the training period. Nevertheless, the training tasks were modified on the basis of the test task. Due to this close resemblance, the participants may have learned not only the targeted skills, but also the task *per se* (e.g., the stimuli, task instructions, and duration of the stimulus array) while performing the training tasks. On the one hand, this characteristic, which was not present in Arend and Zimmer [6], may have contributed to increasing the positive effects on the VWM capacity. On the other hand, it may have limited more extended transfer effects to be produced—for example, a transfer effect in a shape change detection task (although this is a testable matter in the future). Moreover, it would have been helpful for the current study to have included additional assessment tools (e.g., a flanker or attentional blink task) to substantiate whether the filtering training increased general attention control skills and the consolidation training enhanced quick consolidation skills. As our primary focus was to test training-induced benefits on the VWM capacity in a change detection task, we can only draw conclusions about the training effects based on the changes made in the VWM capacity at this time.

Following the recent training studies [7, 11, 14], we used an individually tailored adaptive method. Although the current study did not test the superiority of adaptive training to non-adaptive training methods, it appeared that the individualized adaptive challenges were conducive to producing positive training effects. With this methodology, the two training groups yielded differential results as a function of the set sizes. Unlike the consolidation training, the filtering training affected larger set sizes. This indicated that the impact of the filtering training was broader and more effective than that of the consolidation training for higher memory loads. These findings were made possible from our analysis by set size. Most of the past VWM training studies did not address set size effects, with the exception of two recent studies [6, 8], which examined training effects according to different set sizes and provided more specific results. Likewise, the current study was able to provide more detailed results by examining the set size effects. Lastly, we added the consolidation group to the traditional comparison between an experimental and a control group. The addition of the consolidation group allowed for more rigorous assessment of the training effects, by representing an active control group in which random factors such as the participants’ efforts, motivation, commitment, or expectancy were comparable to those in the filtering group.

The VWM capacity increase shown in the current study could have interesting implications for the capacity debate [2, 28, 29]. However, we do not hold a specific position as to whether VWM capacity is fixed or flexible because (a) our study was not designed to test these models; and (b) the increase shown in this study occurred within a range of 3–4 items. Instead, we wish to highlight the fact that when the training was adaptive, individualized, and targeted, it produced positive effects on the capacity of VWM. Although a number of questions remain to be answered regarding the training effects such as their generalizability or longevity, it is impossible to address those in a single study. With the present research, we think that we have taken a first step toward identifying the important constituents of effective VWM training. We plan to ensure the robustness of these methods for the VWM capacity, and will seek to apply the lessons we have learned to different domains in which effective cognitive interventions are needed (e.g., cognitive aging, autistic child development).
